# Exome Analysis Reveals Differentially Mutated Gene Signatures of Stage, Grade and Subtype in Breast Cancers

**DOI:** 10.1371/journal.pone.0119383

**Published:** 2015-03-24

**Authors:** You Li, Xiaosheng Wang, Suleyman Vural, Nitish K. Mishra, Kenneth H. Cowan, Chittibabu Guda

**Affiliations:** 1 Department of Genetics, Cell Biology and Anatomy, University of Nebraska Medical Center, Omaha, Nebraska, United States of America; 2 Fred and Pamela Buffett Cancer Center, Nebraska Medical Center, Omaha, Nebraska, United States of America; 3 Eppley Institute for Cancer Research, Nebraska Medical Center, Omaha, Nebraska, United States of America; 4 Bioinformatics and Systems Biology Core, University of Nebraska Medical Center, Omaha, Nebraska, United States of America; CNR, ITALY

## Abstract

Breast cancers exhibit highly heterogeneous molecular profiles. Although gene expression profiles have been used to predict the risks and prognostic outcomes of breast cancers, the high variability of gene expression limits its clinical application. In contrast, genetic mutation profiles would be more advantageous than gene expression profiles because genetic mutations can be stably detected and the mutational heterogeneity widely exists in breast cancer genomes. We analyzed 98 breast cancer whole exome samples that were sorted into three subtypes, two grades and two stages. The sum deleterious effect of all mutations in each gene was scored to identify differentially mutated genes (DMGs) for this case-control study. DMGs were corroborated using extensive published knowledge. Functional consequences of deleterious SNVs on protein structure and function were also investigated. Genes such as ERBB2, ESP8, PPP2R4, KIAA0922, SP4, CENPJ, PRCP and SELP that have been experimentally or clinically verified to be tightly associated with breast cancer prognosis are among the DMGs identified in this study. We also identified some genes such as ARL6IP5, RAET1E, and ANO7 that could be crucial for breast cancer development and prognosis. Further, SNVs such as rs1058808, rs2480452, rs61751507, rs79167802, rs11540666, and rs2229437 that potentially influence protein functions are observed at significantly different frequencies in different comparison groups. Protein structure modeling revealed that many non-synonymous SNVs have a deleterious effect on protein stability, structure and function. Mutational profiling at gene- and SNV-level revealed differential patterns within each breast cancer comparison group, and the gene signatures correlate with expected prognostic characteristics of breast cancer classes. Some of the genes and SNVs identified in this study show high promise and are worthy of further investigation by experimental studies.

## Introduction

Breast cancer is the most common cancer (29% of newly diagnosed cancers) in women in US, and has the second highest mortality rate that accounts for about 25% of all cancer deaths [1]. It has been recognized that categorization of breast cancers into different subtypes can effectively guide treatments and greatly improve the prognosis. Several factors like hormone receptor status, breast cancer biomarkers and gene expression profiles have been used to classify breast cancers, estimate the recurrence risk, and guide targeted treatment [2].

Breast cancers are highly heterogeneous in their clinical and molecular profiles, which suggest that the prognosis for each subtype is very distinct. For example, estrogen and progesterone hormone receptor positive (ER+ and PR+) breast cancers have a better prognosis than estrogen and progesterone receptor negative (ER- and PR-) breast cancers. In addition, ER+ and PR+ breast cancers can be treated with anti-hormonal therapy, while ER- and PR- breast cancers are not responsive to such therapies. On the other hand, HER2-positive (HER2+) breast cancers usually occur in younger women, grow more invasively, and prior to the advent of targeted therapy, posed a higher risk of recurrence than HER2-negative (HER2-) breast cancers, partly because of the overexpression of HER2/neu protein (human epidermal growth factor receptor 2, also known as ERBB2) in these cancers.

So far, breast cancer is one of the few cancer types in which targeted therapies have been designed based on the molecular classification [3]. In addition, the gene expression profiling based classification of breast cancers has identified four major subtypes: luminal A, luminal B, human HER2+, and basal-like [4], which have prognostic implications. For example, Oncotype Dx, a 21-gene assay [5], and Mammaprint, a 70-gene expression signature have been developed as a prognostic assessment tool to predict the risk of breast cancer metastasis [6]. However, one disadvantage of using gene expression profiling to identify biomarkers or signatures for cancer is that gene expression levels are highly variable and unsteady, and therefore a single measure often leads to misinterpretation. In contrast, genetic mutations at DNA level can be stably detected. As all cancers carry somatic mutations in their genomes and mutational heterogeneity widely exists in cancer genomes [7], biomarkers for cancer based on gene mutation information could be detected more accurately than those based on gene expression profiling. Rapid advances in next-generation sequencing (NGS) technology have enabled sequencing of a large number of whole exome samples in parallel at a reasonable expense. As a result, a large amount of NGS data on tumor genomes have emerged that makes detection and application of genomic mutant-based biomarkers for cancer a reality.

While differential gene expression among different subtypes of breast cancer have been widely used for assessing prognosis and predicting therapeutic response [8], The Cancer Genome Atlas (TCGA) network analyzed differential somatic mutations among the four breast cancer subtypes: luminal A, luminal B, HER2+, and basal like, and identified several significantly mutated genes that showed subtype-specific patterns of mutation [9]. Some of the studies report specific DNA mutations from comparisons of ER+/- [10] or HER2+/- classes [11], simply by checking genes that encode ER (ESR1 and ESR2) and HER2 (ERBB2), respectively. However, no systematic studies have been carried out to identify DMGs between the ER, PR, HER2 subtypes, or the tumor grade and stage classes. In the present study, we analyzed 98 breast cancer exome sequencing datasets that were previously published [12]. We performed large-scale comparison of single nucleotide variation (SNV) differences between three breast cancer subtypes (ER+ vs. ER-, PR+ vs. PR-, HER2+ vs. HER2-), two different histologic grades (grade II vs. grade III), and two different stages (stage II vs. stage III), all of which are clinical features that are directly associated with prognosis of breast cancers. We did not use PAM50 or other gene-expression based subtypes for identifying DMGs because there is no evidence showing that gene expression profiles are directly correlated with gene mutation profiles. We proposed a scoring function to evaluate the deleterious impact of the sum of all mutations in a gene, and then used multiple t-tests to identify DMGs between the five breast cancer comparison groups described above. We performed an extensive examination of literature to confirm the relevance of the identified DMGs to breast or other cancers. We also identified the deleterious SNVs from the DMGs that occur with significantly different frequency in between the five breast cancer comparison groups. For some important mutations, we also examined the impact of each mutation on the structure and function of the protein using protein-modeling tools.

## Materials and Methods

### Breast cancer whole exome-seq datasets

We downloaded the whole exome sequencing datasets for 103 breast cancer samples (54 samples from Mexican patients and 49 samples from Vietnamese patients) from dbGap website http://www.ncbi.nlm.nih.gov/gap (accession number: phs000369.v1.p1) [12]. In this study, we analyzed only 98 samples because 5 Mexican samples have very low sequencing quality. All the 98 breast cancer samples contain tumor/normal pairs. We assume that germline and acquired somatic mutations (till the diagnosis of cancer) could significantly contribute to the differential phenotype of breast cancers [13]; hence, we did not filter out the mutations that are present in the normal sample. Based on the clinical information provided, we sorted the 98 samples (patients) into five comparison groups that include three clinical subtype groups (ER+ vs. ER-, PR+ vs. PR-, HER2+ vs. HER2-), a grade-based (grade II vs. grade III), and a stage-based (stage II vs. stage III) group. A summary of the classification information for the 98 samples is shown in Table 1 (Stage I and IV, grade I were excluded in the comparisons due to lack of sufficient data). The mutation profile for each patient is often sparse. When comparing one class with smaller sample size (<10) against another class with a larger sample size (i.e. >20) in another, one mutation in the former class will be considered to occur at a rate of more than 10%. Therefore, we set a cut off value of 10 to define the descent sample size, in order to minimize the impact of rare mutations in classes with smaller sample size in the statistical tests. In this case, any class that has less than 10 samples will not be compared separately, if applicable (Table 1). We also performed Fisher’s exact test to check the race compositional differences between each comparison group. Notably, the race composition in grade II and grade III is unbalanced (Table 1 and S1 Table); therefore, we only performed the class comparison for Mexican patients, in order to eliminate the effect of any race-specific genetic variations. (A detailed description of clinical information for all samples is shown in S1 Table).

**Table 1:** A summary of the five comparison groups of breast cancers used in this study.

**Class**	**ER+**	**ER-**	**PR+**	**PR-**	**HER2+**	**HER2-**	**GradeII**	**GradeIII**	**StageII**	**StageIII**
**MEX**	35	14	31	18	8^a^	41	25	13	32	10
**VIE**	5^a^	13	6^a^	12	0^a^	1^a^	0^a^	13^c^	38	8^a^
**P (Fisher's Exact Test)**	0.001892^b^		0.05079		1		1^c^		0.598	
**Sample used in this Study**	40	27	37	30	8	42	25	13	70	18

^a^ Sample size for these class are too small (<10) for separate class comparison among each race.

^b^ Fisher’s exact tests have been conducted in order to check the distribution difference of Mexican and Vietnam patients in each comparison group. Only ER comparison group has significantly different race composition (p<0.05).

^c^ 25 of the patients in Grade II are all Mexican patients, compared to 13 Mexican patients and 13 Vietnamese Patients in Grade III. Therefore, we excluded 13 Samples from Vietnam Grade III patients and the sample sizes of Grade II vs. Grade III used in this study (all Mexican patients), are 25 and 13 respectively. The reported fisher’s exact test statistics for this comparison group is also based on the exclusion of Vietnam patient samples.

### Sample quality control, alignment, SNV calling and annotation

We used FastQC [14] and FastX toolkit [15] for quality control of the 98 tumor whole exome sequencing datasets. Short reads with low sequencing quality (Phred score < 20) were removed or trimmed, accordingly. Processed reads were then aligned with Borrows-Wheeler Aligner [16] to the human reference genome hg19. We then applied the Genome Analysis Toolkit [17] (GATK) best practices pipeline [18,19] from Broad Institute for SNV (Single Nucleotide Variant) calling from alignment files, and the pipeline includes multiple steps such as Mark Duplicates, Local Realignment, Quality Score Recalibration and variant calling. After 98 SNV profiles were generated, we used ANNOVAR [20] for functional annotation of all the SNVs. The SIFT [21] score reported from ANNOVAR was used to evaluate the degree of deleteriousness of SNVs.

### Scoring the deleteriousness of mutated genes

The SIFT score ranges from 0 to 1. An SNV is predicted to be deleterious when its SIFT score is less than or equal to 0.05. Therefore, we filtered out all the SNVs that have the SIFT score more than 0.05. We calculated the deleterious score, *D*, using the following function,

$$D_{ij}=\sum_{k}\left(\frac{\sum_{x}\left(1-S_{ijkx}\right)}{N_{k}}\right),$$

Where

*D*_*ij*_: the deleterious mutation score for the *i* ^th^ gene in sample *j*;

*S*_*ijkx*_: the SIFT score for the *k* ^th^ mutation in isoform *x* of the *i* ^th^ gene in sample *j*;

*N*_*k*_: the number of isoforms that are affected by the mutation *k* for that specific gene in that sample.

This scoring function combines the SIFT scores for all deleterious mutations in a gene (including the isoforms, if any) and generates a combined deleterious score for each mutated gene. Therefore, by applying this scoring function, we obtained a matrix with 98 columns (98 patients) and about 17000 rows (~17000 RefSeq genes). Each cell represents how deleterious one gene is mutated for the specific patient. Obviously, the higher the score is, the more deleterious way the gene mutations affect the gene function.

### Identification of DMGs between breast cancer classes

We identified DMGs between five pairs of breast cancer class comparisons (ER+ vs. ER-, PR+ vs. PR-, HER2+ vs. HER2-, grade II vs. grade III, and stage II vs. stage III) using the univariate t-test at a two-sided significance level of 0.001. Considering the small sample size of grade I and stage I classes, we only performed grade II vs. grade III, and stage II vs. stage III class comparisons (no patients with stage IV breast cancer were present in our list). To adjust for multiple testing, we also reported the false discovery rate (FDR) for each gene identified. The FDR was estimated using the method of Benjamin and Hochberg [22]. This procedure was implemented with the class comparison tool in BRB-ArrayTools [23].

### Functional analysis of DMGs and SNVs

We examined the deleterious SNVs present in the DMGs of different breast cancer classes using an odd ratio, which identifies SNVs that are at least 2-fold more frequent in one class over the other between the populations of a two-class comparison. Fisher’s exact test was used to examine the significance of the differences. We then used Pfam (protein family) database [24] and CONDEL [25] to predict the functional impact of those significant SNVs on proteins. Pfam database contains information on evolutionarily conserved functional domains; hence, if an SNV occurs in the domain region, it is more likely to affect the structure and/or function of the protein. CONDEL is a method to assess the outcome of non-synonymous SNVs with the best sensitivity and specificity [25]. It uses the consensus deleterious scores by combining predictions from five different tools that include SIFT[21], PolyPhen2[26], Logre[27], MutationAssessor[27] and MAPP[28].

### Protein stability analysis for point mutations

For the DMGs, we analyzed the overall impact of point mutations on protein stability. For feasibility of analysis, we selected a set of 10 relatively rare non-synonymous SNVs that occur either in a functionally annotated (Pfam-A) or evolutionarily conserved (Pfam-B) domain region. We used iprscan version 4.8 [29] for Pfam and PANTHER motif search. We then used two reliable structure prediction tools, RaptorX [30] online webserver and I-TASSER suite [31] standalone version, for protein structure prediction. We ran I-TASSER in parallel mode with the default parameters.

Further, we used three similar and independent tools, I-Mutant-2.0 [32], PopMusic-2.1 [33] and CUPSAT [34] to analyze the overall impact of a point mutation on protein stability. I-Mutant predicts the stability of a point-mutated protein from its primary sequence, while PopMusic 2.1 and CUPSAT predict the same from its 3D structure. We evaluated the overall impact of a point mutation on protein stability based on the consensus results from these three methods; if at least two tools predict the same mutation effect on the protein structure, i.e., destabilizing or stabilizing, then only we accept that result.

## Results and Discussion

Samples in ER, PR, HER2 and Stage are all having at least one class with the sample size of less than 10, if we separate each class by race (Table 1). Therefore, we merged all the samples that are available for each class. Notably, giving the clinical significance of HER2 status in breast cancer, we still performed the class comparison for HER2 group, despite the fact that HER2+ class only has 8 samples. For Grade II vs. Grade III, we excluded all the Vietnam patients, as the unbalanced sample size in Vietnam patients (0 samples in Grade II and 13 samples in Grade III) will definitely bias the test result. Fisher’s exact test result from table 1 shows that only ER class has significantly different race composition (p = 0.0018). This could indicate the potential impact of the race factor on ER comparison group result. However, the statistical power of the comparison will be limited by the number of sample size, if we do the ER comparison for each race separately.

SNV profiles were generated for 98 tumor exome-seq datasets using the GATK pipeline, and annotated using ANNOVAR. A mutation score matrix was created for all 23,769 RefSeq genes (42,239 transcripts in total) in all the samples based on the annotation results. We filtered out those genes that have deleterious mutations present in less than 5 (out of 98) samples, and obtained 3,826 genes for further analysis (S2 Table). Combined mutation score (from all mutations of all isoforms of a gene) of each gene was compared between the defined breast cancer classes to identify sets of DMGs. These include 18 (ER+ vs. ER-), 9 (PR+ vs. PR-), 10 (HER2+ vs. HER2-), 10 (grade II vs. grade III) and 7 genes (stage II vs. stage III), using a two-sided t-test (p-value≤0.001).

**Fig 1:**
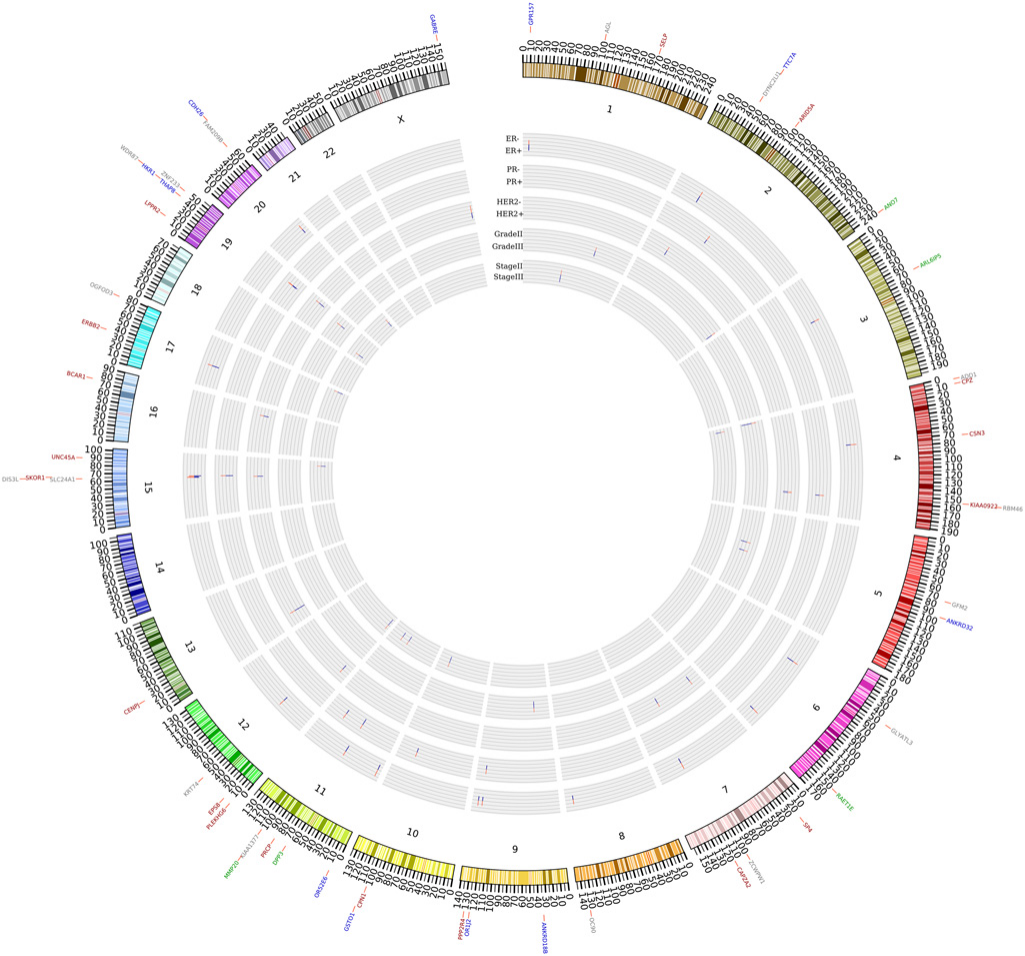
The differentially mutated genes between breast cancer subtypes. A total of 50 genes are identified that are differentially mutated by comparison of ER+ vs. ER-, PR+ vs. PR-, HER2+ vs. HER2-, grade II vs. grade III, and stage II vs. stage III breast cancer classes, respectively. Each class comparison is shown in layered circles. The differentially mutated genes are shown in the outer layer, which correspond to their chromosome coordinates and subtype comparisons. The differentially mutated genes are sorted into four different categories based on their relevance to breast cancer or other types of cancer. Category 1 includes the genes that are directly related to breast cancer (in dark red). Category 2 includes the genes that are related to other types of cancer (in green). Category 3 includes the genes whose family members are related to cancer (in blue). Category 4 includes the genes whose relatedness to cancer remains to be studied (in gray). The mean deleterious mutation score for each gene in each class comparison is shown in colored thin bar (red and blue colors refer to two different classes). The length of thin bars is proportional to the mean deleterious score.

Using literature survey, we sorted the DMGs into 4 different categories in the order of their relevance to breast cancer or other types of cancers. Category 1 includes the genes that have been reported to be directly associated to breast cancer, while category 2 includes those that are related to other types of cancer, but not to breast cancer. Category 3 includes the genes whose functions have not been well studied, but other members of these gene families have been reported to be associated to cancer. Category 4 includes the other genes that do not belong to the former three categories, while their relatedness to cancer remains to be studied. Fig. 1 presents the CIRCOS [35] graph of the DMGs identified by the five class comparisons along with their corresponding chromosomal positions. It shows that chromosome 4, 11, and 19 have the largest number (5 genes in each chromosome) of DMGs identified in the comparisons, while chromosome 14, 18, 21, and 22 do not contain any of the reported DMGs. Also, chromosome 4 has the largest number of DMGs (CPZ, CSN3, KIAA0922) that are directly related to breast cancer. Moreover, because of the similarities of ER and PR status in terms of breast cancer prognosis and therapy, the positional pattern for ER+/- and PR+/- is similar by having the same 3 DMGs in both group comparisons.

**Fig 2:**
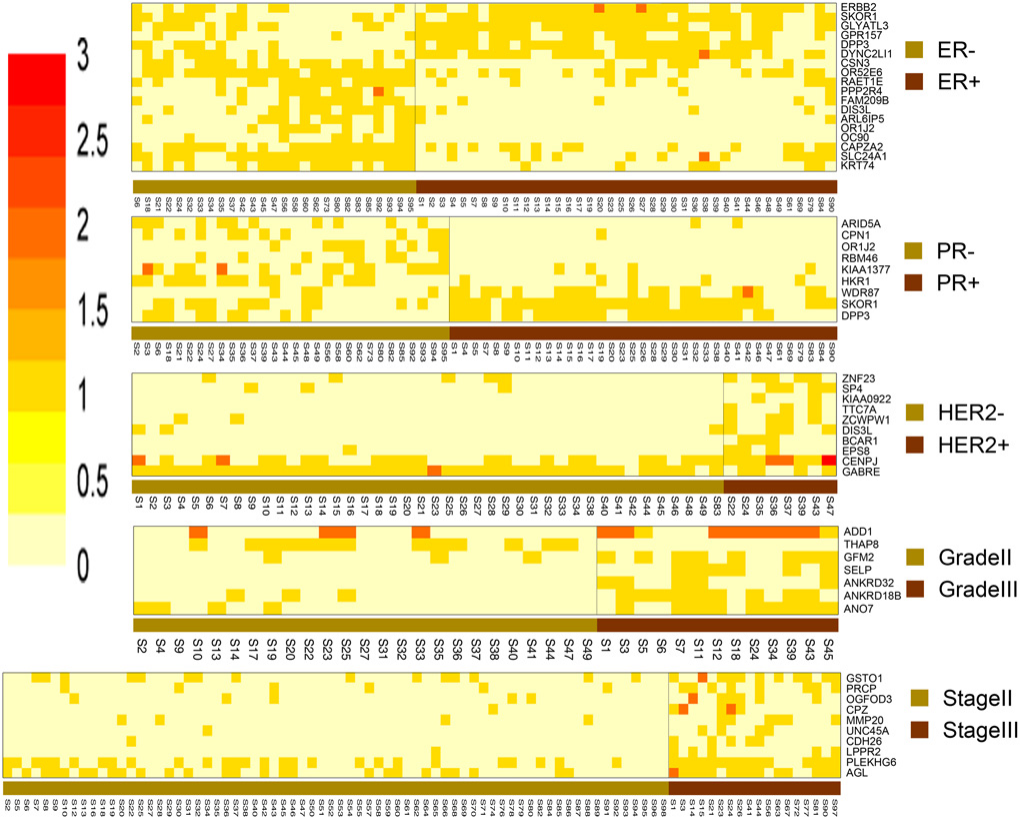
The deleterious mutation scores for the differentially mutated genes across the compared samples. Five heatmaps show the deleterious mutation scores across the compared samples for the differentially mutated genes identified by comparison of ER+ vs. ER-, PR+ vs. PR-, HER2+ vs. HER2-, grade II vs. grade III, and stage II vs. stage III breast cancer classes, respectively. Higher score implies more deleterious mutations one gene has. It is evident that groups with better prognosis (ER+, PR+, HER2-, Stage II and Grade II) tend to have fewer deleteriously mutated genes.

Fig. 2 is a heatmap showing the deleterious mutation patterns for DMGs identified by five groups of breast cancer class comparisons. It is evident that the overall deleterious mutation scores are higher in classes with poorer prognosis (ER-, PR-, HER2+, Grade III and Stage III), suggesting that deleterious mutations in these genes contribute to different prognostic features for each class. However, it is also evident that sets of genes within a class comparison show contrasting deleterious mutation patterns suggesting that their roles as oncogenes or tumor suppressor gene are balanced (Fig. 2). For instance, ERBB2, an oncogene is predominantly mutated in ER+ class (77.5%) compared to ER- class (33.3%), suggesting that dysregulation or altered function of HER2/neu protein is associated with a better prognosis in breast cancer patients. In contrast, CSN3, a part of the CSN complex that activates tumor suppressor TP53, is predominantly less frequently mutated in ER+ (17.5%) compared to ER- (63.0%) samples. Descriptive information for all the identified DMGs is presented in Tables 2–6 and in the S1 File.

**Table 2:** Differentially mutated genes between ER+ and ER- breast cancer subtypes.

**Gene Symbol**	**P-value**	**FDR** ^a^	**Mean of mutation score in ER-**	**Mean of mutation score in ER+**	**FC** ^b^	**Gene Name**	**Category** ^c^
CSN3	7.06E-05	0.090	0.61	0.16	3.75	casein kappa	1
ERBB2	1.57E-04	0.099	0.32	0.81	0.40	v-erb-b2 erythroblastic leukemia viral oncogene homolog 2, neuro/glioblastoma derived oncogene homolog (avian)	1
PPP2R4	2.09E-04	0.099	0.44	0.04	10.40	protein phosphatase 2A activator, regulatory subunit 4	1
CAPZA2	4.02E-04	0.128	0.75	0.33	2.24	capping protein (actin filament) muscle Z-line, alpha 2	1
SKOR1	7.56E-04	0.181	0.41	0.80	0.51	SKI family transcriptional corepressor 1	1
ARL6IP5	1.72E-04	0.099	0.40	0.04	9.39	ADP-ribosylation-like factor 6 interacting protein 5	2
RAET1E	2.28E-04	0.099	0.63	0.20	3.14	retinoic acid early transcript 1E	2
DPP3	2.54E-04	0.099	0.26	0.70	0.38	dipeptidyl-peptidase 3	2
OR1J2	4.04E-05	0.090	0.33	0.00	INF	olfactory receptor, family 1, subfamily J, member 2	3
OR52E6	1.68E-04	0.099	0.86	0.43	2.00	olfactory receptor, family 52, subfamily E, member 6	3
GPR157	5.57E-04	0.142	0.18	0.58	0.30	G protein-coupled receptor 157	3
SLC24A1	6.30E-05	0.090	0.85	0.34	2.46	solute carrier family 24 (sodium/potassium/calcium exchanger), member 1	4
KRT74	2.59E-04	0.099	0.59	0.18	3.37	keratin 74	4
DIS3L	2.85E-04	0.099	0.49	0.10	4.97	DIS3 mitotic control homolog (S. cerevisiae)-like	4
OC90	4.68E-04	0.138	0.26	0.00	INF	otoconin 90	4
DYNC2LI1	5.56E-04	0.142	0.37	0.80	0.46	dynein, cytoplasmic 2, light intermediate chain 1	4
GLYATL3	8.67E-04	0.192	0.44	0.82	0.54	chromosome 6 open reading frame 140	4
FAM209B	9.02E-04	0.192	0.43	0.10	4.44	family with sequence similarity 209, member B	4

^a^ FDR: False Discovery Rate

^b^ FC: fold change (ER-/ER+); INF: infinite

^c^ Category 1: directly related to breast cancer;

Category 2: related to other types of cancer, but not to breast cancer;

Category 3: other members of the same family (but not by itself) are related to cancer;

Category 4: not belonging to any of the former three categories.

*All the above notations apply to Tables 3, 4, 5 and 6.

**Table 3:** Differentially mutated genes between PR+ and PR- breast cancer subtypes.

Gene Symbol	p-value	FDR ^a^	Mean of mutation score in PR-	Mean of mutation score in PR+	FC ^b^	Gene Name	Category ^c^
SKOR1	1.14E-04	0.297	0.40	0.84	0.48	SKI family transcriptional corepressor 1	1
CPN1	5.47E-04	0.425	0.32	0.03	11.27	carboxypeptidase N, polypeptide 1	1
ARID5A	1.00E-03	0.425	0.37	0.06	6.49	AT rich interactive domain 5A (MRF1-like)	1
DPP3	7.74E-04	0.425	0.30	0.70	0.42	dipeptidyl-peptidase 3	2
OR1J2	2.14E-04	0.297	0.30	0.00	INF	olfactory receptor, family 1, subfamily J, member 2	3
HKR1	8.95E-04	0.425	0.50	0.14	3.60	GLI-Kruppel family member HKR1	3
KIAA1377	2.33E-04	0.297	0.56	0.11	5.01	KIAA1377	4
RBM46	5.91E-04	0.425	0.26	0.00	INF	RNA binding motif protein 46	4
WDR87	8.72E-04	0.425	0.14	0.54	0.26	WD repeat domain 87	4

*All the notations are the same as in Table 2.

**Table 4:** Differentially mutated genes between HER2+ and HER2- breast cancer subtypes.

Gene Symbol	p-value	FDR ^a^	Mean of mutation score in HER2-	Mean of mutation score in HER2+	FC ^b^	Gene Name	Category ^c^
BCAR1	1.06E-05	0.020	0.00	0.37	0.00	similar to breast cancer anti-estrogen resistance 1; breast cancer anti-estrogen resistance 1	1
CENPJ	2.59E-04	0.248	0.56	1.46	0.38	centromere protein J	1
EPS8	4.61E-04	0.294	0.03	0.37	0.08	epidermal growth factor receptor pathway substrate 8	1
KIAA0922	6.15E-04	0.332	0.00	0.25	0.00	KIAA0922	1
SP4	9.61E-04	0.380	0.07	0.48	0.15	Sp4 transcription factor	1
GABRE	3.89E-04	0.294	0.93	0.48	1.95	gamma-aminobutyric acid (GABA) A receptor, epsilon	3
TTC7A	1.06E-05	0.020	0.00	0.38	0.00	tetratricopeptide repeat domain 7A	3
DIS3L	1.00E-03	0.380	0.07	0.50	0.14	DIS3 mitotic control homolog (S. cerevisiae)-like	4
ZCWPW1	1.39E-04	0.177	0.04	0.49	0.09	zinc finger, CW type with PWWP domain 1	4
ZNF233	6.95E-04	0.332	0.12	0.62	0.20	zinc finger protein 233	4

*All the notations are the same as in Table 2.

**Table 5:** Differentially mutated genes between Grade II and Grade III breast cancer classes.

Gene Symbol	p-value	FDR ^a^	Mean of mutation score in Grade II	Mean of mutation score in Grade III	FC ^b^	Gene Name	Category ^c^
SELP	6.73E-05	0.230	0.00	0.45	0.00	selectin P (granule membrane protein 140kDa, antigen CD62)	1
ANO7	6.32E-04	0.460	0.16	0.68	0.24	anoctamin 7	2
ANKRD18B	1.22E-04	0.230	0.12	0.69	0.18	ankyrin repeat domain 18B	3
ANKRD32	4.70E-04	0.450	0.00	0.38	0.00	ankyrin repeat domain 32	3
THAP8	8.19E-04	0.460	0.51	0.00	INF	THAP domain containing 8	3
ADD1	3.63E-04	0.450	0.31	1.33	0.23	adducin 1 (alpha)	4
GFM2	8.37E-04	0.460	0.12	0.61	0.20	G elongation factor, mitochondrial 2	4

*All the notations are the same as in Table 2.

**Table 6:** Differentially mutated genes between Stage II and Stage III breast cancer classes.

Gene Symbol	p-value	FDR ^a^	Mean of mutation score in Stage II	Mean of mutation score in Stage III	FC ^b^	Gene Name	Category ^c^
CPZ	4.24E-05	0.055	0.01	0.39	0.04	carboxypeptidase Z	1
LPPR2	4.29E-05	0.055	0.01	0.26	0.05	lipid phosphate phosphatase-related protein type 2	1
PRCP	1.44E-04	0.138	0.08	0.43	0.19	prolylcarboxypeptidase (angiotensinase C)	1
UNC45A	5.03E-04	0.297	0.01	0.23	0.06	unc-45 homolog A (C. elegans)	1
PLEKHG6	5.44E-04	0.297	0.38	0.82	0.46	pleckstrin homology domain containing, family G (with RhoGef domain) member 6	1
MMP20	7.99E-04	0.339	0.06	0.33	0.17	matrix metallopeptidase 20	2
CDH26	4.21E-05	0.055	0.01	0.28	0.05	cadherin-like 26	3
GSTO1	2.93E-04	0.224	0.21	0.66	0.32	glutathione S-transferase omega 1	3
AGL	6.42E-04	0.307	0.37	0.83	0.44	amylo-1, 6-glucosidase, 4-alpha-glucanotransferase	4
OGFOD3	9.77E-04	0.374	0.07	0.39	0.18	2-oxoglutarate and iron-dependent oxygenase domain containing 3	4

*All the notations are the same as in Table 2.

Notably, some of the FDR values we reported in Tables 2–6 are relatively high. This is because in the present study, we only considered SNVs whose SIFT score is not greater than 0.05. As a result, our deleterious mutation scores lie in a relatively narrow range, which could have generated high FDR values.

### Comparison of DMGs in hormone receptor positive vs. negative breast cancer subtypes

Due to many similarities between the ER+/- and PR+/- class comparisons, we are presenting these two classes together in this section. We identified 18 genes with significantly different mutation scores between ER+ and ER- (Fig. 2, Table 2), and 9 genes between PR+ and PR- breast cancer subtypes (Fig. 2, Table 3). Three genes, OR1J2, SKOR1, and DPP3 are commonly identified in both class comparisons. In Tables 2 and 3, genes are listed based on their biological relevance to breast cancer. Of these, CSN3, ERBB2, PPP2R4, CAPZA2, SKOR1, ARID5A, and CPN1 belong to category 1 that contain literature-based relevance to breast cancer. Below, we describe the functional roles of all DMGs under category 1 in each comparison group, while description of all other DMGs can be found in S1 File.

CSN3 (kappa-casein) is involved in myeloid leukemia factor 1-mediated growth arrest and CSN3 deficiency impairs p53 activation, facilitates cell proliferation and affects COP1-mediated p53 degradation [36]. It indicates that mutationally impaired CSN3 could promote cancer growth and progression by dysregulation of the tumor suppressor gene p53. This is consistent with our results that CSN3 has a higher deleterious mutation score in the more aggressive ER- breast cancers compared to that in less aggressive ER+ breast cancers (p-value = 7.06×10^-5^).

On the other hand, ERBB2 (also known as HER2/neu), is a well-characterized oncogene that is responsible for development and progression of certain aggressive types of breast cancer. ERBB2 has been shown to be associated with poor prognosis of breast cancers [37]. Overexpression of this gene has been shown to be very crucial in the development and progression of certain aggressive types of breast cancer [38]. Our results corroborate that ERBB2 shows higher mutational load (p-value = 1.57×10^-4^) in ER+ breast cancers compared to ER- breast cancers and consequently dysregulated to negate cancer growth and progression in the former subtype.

PPP2R4, also known as protein phosphatase 2A (PP2A), regulates estrogen receptor alpha (ER-α) expression through modulation of ER mRNA stability; hence, it has been considered as a potential therapeutic target for breast cancer [39]. It has been shown that PPP2R4 is involved in PI3K/Akt signaling pathway, a pathway that modulates the interaction between BRCA1 and ER-α [40]. Mutations of PPP2R4 have been shown to contribute to many cancer types including breast cancers [41], and it has been suggested that PPP2R4 might be a tumor suppressor gene [42]. Our results show that PPP2R4 has more deleterious mutations in ER- breast cancers than in ER+ breast cancers (p-value = 2.09×10^-4^), suggesting that the higher degree of loss of tumor suppression function for PPP2R4 in ER- subtype relative to ER+ contributes to worse prognosis in the former.

CAPZA2, named as F-actin-capping protein subunit alpha-2, is regulated by Erbb2 in mouse model [43]. It may be also involved in human Ras-MAPK/P13K signaling pathways, as it is predicted to interact with a retinoblastoma tumor suppressor (pRB) protein [44]. Consistent with this notion, our results show that this gene has a higher deleterious mutation score in ER- breast cancers than in ER+ breast cancers (p-value = 4.02×10^-4^).

SKOR1, also known as Fussel-15, is a SKI family transcriptional co-repressor that is identified as a DMG both in ER+/- and PR+/- comparisons. It is also a potential repressor of the BMP signaling pathway [45]. A previous study shows that repressing BMP signaling pathway can efficiently prevent bone metastasis from breast cancer cells [46]. Our results show that the gene has a higher deleterious mutation score in ER+ breast cancers relative to ER- breast cancers (p-value = 7.56×10^-4^). Similarly, this gene has a higher deleterious mutation score in PR+ breast cancers than in PR- breast cancers (p-value = 1.14×10^-4^).

ARID5A has been identified as an ER-α interacting co-repressor protein. ARID5A represses transcriptional activity of endogenous ER-α in MCF-7 breast cancer cells [47]. This gene has a higher deleterious mutation score in PR- breast cancers than in PR+ breast cancers (p-value = 1.0×10^-3^).

CPN1 gene encodes an enzyme that is responsible for C-terminal cleavage of stromal cell derived factor-1α (SDF-1) [48]. SDF-1 functions as a growth factor for immature B-lymphocytes and controls chemokine expression, thereby regulating the destination of metastasizing breast cancer cells [49]. Besides, studies show that CPN1 is an estrogen target gene in zebrafish model [50]. This gene has a higher deleterious mutation score in PR- breast cancers than in PR+ breast cancers (p-value = 5.47×10^-4^).

### Comparison of DMGs in HER2+ vs. HER2- breast cancer subtypes

We identified 10 genes that have significantly different deleterious mutation scores between HER2+ and HER2- breast cancer subtypes as listed in Table 4 (Fig. 2). Among them, BCAR1, CENPJ, EPS8, KIAA0922, and SP4 are directly related to breast cancer as described below. Literature information for Category 2–4 genes can be found in the S1 File.

BCAR1 is a breast cancer anti-estrogen resistance kinase. Previous studies showed that BCAR1 is responsible for resistance to the anti-proliferative effects of tamoxifen [51,52] and its expression level often positively correlate with ERBB2 expression [53], thereby leading to aggressive tumor progression. Table 4 shows that more deleterious mutations of BCAR1 were detected in HER2+ than in HER2- breast cancer subtypes (p-value = 1.06×10^-5^), suggesting that BCAR1 mutations lead to its hyperactivation that correlates with the overexpression of ERBB2. Interestingly, it has been found that higher BCAR1 levels were significantly associated with ER+/PR+ tumors [54].

CENPJ encodes centromere protein J that is a co-activator for STAT5 signaling pathway [55] and NF-kappa-B-mediated transcription [56]. Nuclear localization of STAT5 marks a good prognosis of ER+/PR+ breast cancers [57] and could be used as an indicator of anti-estrogen therapy [58]. NF-kappa-B pathway may be involved in the gain of resistance to HER2- targeting agents therapy [59]. Our results suggest that mutations in CENPJ could potentially be the driver events as the deleterious mutation score for CENPJ in HER2+ breast cancers is much higher than that in HER2- breast cancers (p-value = 2.59×10^-4^).

EPS8, an epidermal growth factor receptor pathway substrate 8, has been identified as a novel candidate oncogene for breast cancer [60]. EPS8 also decreases chemosensitivity and affects survival of cervical cancer patients [61]. It has been found that small interfering RNA of Eps8, could reduce proliferation and tumorigenesis in Eps8-attenuated HeLa and SiHa cells cultured in dishes or inoculated in mice [61]. Table 4 shows that EPS8 has higher deleterious mutation score in HER2+ breast cancers than in HER2- breast cancers (p-value = 4.61×10^-4^), suggesting that its mutations might result in poor prognosis of breast cancers.

KIAA0922 is a novel gene detected in Kazusa cDNA sequencing project [62]. Recent studies on KIAA0922 show that it is a transmembrane 131-like (TMEM131L) protein and it functions as a novel regulator of thymocyte proliferation [63]. KIAA0922 also functions as a novel inhibitor of Wnt signaling pathway [63]. Abnormality of Wnt signaling pathway has been associated with breast cancer [64].

Lastly, SP4 is a transcription factor and down-regulation of this gene is associated with inhibited growth of cancer cells in pancreatic [65], colon [66] and breast cancers [67,68].

### Comparison of DMGs in Grade II vs. Grade III breast cancer classes

We identified 7 DMGs between Grade II and Grade III breast cancer subtypes as are listed in Table 5 (Fig. 2). SELP is directly associated with breast cancer [69,70,71]. ANO7 belongs to category 2, and ANKRD18B, ANKRD32 and THAP8 belong to category 3. ADD1 is related to hypertension and SNVs in ADD1 is strongly linked with cancer, but there is no literature evidence showing the involvement of tumorigenesis for this gene. Literature information for Category 2–4 genes can be found in S1 File.

SELP has been a part of an invasive ductal carcinoma gene signature [69]. SELP mediates adhesions for various cells including cancer cells in inflammation, thrombosis, cancer growth and metastasis [70]. High expression of SELP correlates with worse prognosis of human cancer by promoting metastasis of the cancer cells [71].

**Fig 3:**
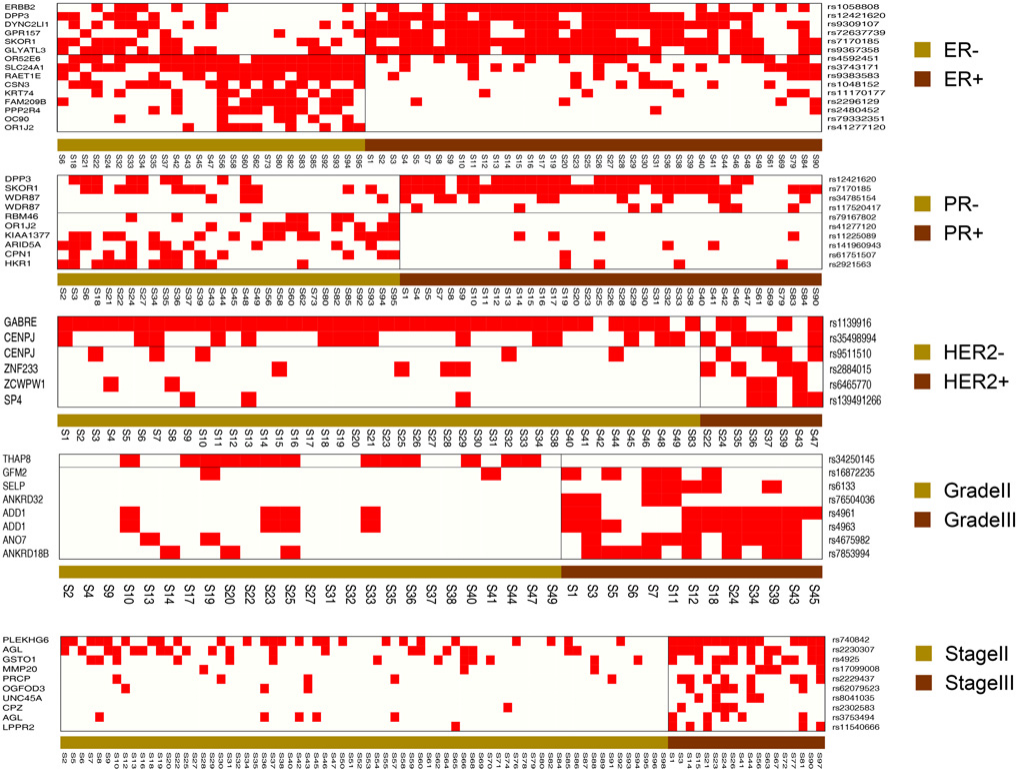
The distribution of deleterious SNVs across the compared patient samples. Five charts illustrate the deleterious mutation distribution in different breast cancer class. Red dot indicates the presence of SNV for the corresponding gene in each sample.

Although there is no direct evidence for the role of ADD1 in breast cancer progression and tumorigenesis, ADD1 has a significantly higher deleterious mutation score in Grade III breast cancers than in Grade II type (p-value = 3.63×10^-4^). Among all patients with grade II and grade III breast cancer, 14 patients have deleterious mutations (rs4961 and/or rs4963) in ADD1, 12 of those have both rs4961 and rs4963 (Fig. 3). A previous study has shown that the carriers of rs4961 were at 1.8 times increased risk for hypertension (CI: 1.32–2.43) [72]. Also, it has been confirmed that rs4963 is tightly linked with rs4961, and thus could also be linked to hypertension [73]. Hypertension has been shown to be one of the common comorbidities in breast cancer patients, and be associated with worse prognosis of breast cancers [74]. Our data shows that 76.9% (10/13) of the grade III breast cancer patients have either rs4961 and rs4963, indicating an increased risk of having hypertension, compared to 16% (4/25) of that for grade II breast cancer patients (Odd ratio is 17.5). Thus, the correlation between hypertension and breast cancer is worth investigating.

It should also be noted that almost all the DMGs between grade II and grade III classes have higher deleterious mutation scores in grade III except one gene (THAP8). This suggests that deleterious gene mutations evolve with the progression of cancer.

### Comparison of DMGs in Stage II vs. Stage III breast cancer classes

We identified 10 DMGs between Stage II and Stage III breast cancer classes as are listed in Table 6. Similar to the grade class, all the genes in Table 6 display higher deleterious mutations in the worse prognosis class (stage III) supporting the general notion that higher mutational load leads to worse prognosis. Half of these genes are directly related to breast cancer (CPZ, LPPR2, PRCP, UNC45A, and PLEKHG6), MMP20 is related to other types of cancer, while CDH26 and GSTO1 belong to category 3. Literature information for category 2–4 genes can be found in S1 File.

CPZ encodes a member of the metallocarboxypeptidase family. This gene is involved in Wnt signaling pathway [75], and therefore potentially plays a role in prognosis of breast cancer. LPPR2 encodes a lipid phosphate phosphatase-related protein that regulates lysophosphatidic acid (LPA) production and signaling [76], and could promote breast cancer initiation, progression and metastasis [77,78,79]. PRCP encodes a protein that acts as a regulator of cell proliferation and autophagy [80], and is also an anti-estrogen resistant protein in ER-positive breast cancer patients [80]. Autophagy functions as a tumor suppressor mechanism, thereby preventing tumor progression [81]. UNC45A encodes a protein that plays a role in cell proliferation and myoblast fusion, and could increase human breast cancer metastasis [82]. Knockdown of UNC45A mRNA slows down human breast carcinoma cell proliferation and invasion [82]. PLEKHG6 regulates the invasion activity of breast cancer cells [83,84].

Based on the five class comparisons we made in this study, it should be noted that there are several potential limitations in this study. First, results from class comparisons with small sample size were more likely to be affected by rare mutations. Secondly, tumor heterogeneity remains a big challenge for SNV analysis, although tumor heterogeneity did not introduce many false positives in this study. Here, we only reported the most likely genotypes using the GATK tool UnifiedGenotyper. Therefore, any reported deleterious mutations should have decent allele frequency in our samples. Heterogeneity of cancer cells would only neutralize the ability to identify those mutations with lower allele frequency. On the other side, a reported deleterious mutation should be either presented in all subclones, or in one or more subclones that are the dominant population in the sample. Thus, our statistical tests only identified the dominant mutations that are more deleteriously mutated in one group compared to another one. To resolve the tumor heterogeneity issue, the single-cell sequencing technology is a good choice.

### Functional analysis of deleterious SNVs

We identified 24 deleterious SNVs that have more than 2-fold difference in the odd ratio while also located inside the functional or conserved domain regions of proteins, from the 117 DMG-associated SNVs (S3 Table). These SNVs are presented in Table 7 (rs12421620 from DPP3 is present in both ER and PR class comparisons). Fisher’s exact tests show that all the odd ratio differences are significant (p≤0.05). For each SNV, we also determined a score that suggests the degree of mutation deleteriousness using the CONDEL software (S4 Table). Fig. 3 shows the presence or absence of SNVs in patients from the five comparison groups of breast cancer. For each class comparison, the frequencies of mutations are highly correlated with the prognostic features. Also, except for the Stage class, all the other classes show contrasting patterns of SNVs (between better and worse prognoses), within each class suggesting their enhancing or suppressing roles in cancer progression. Nine SNVs from ER- vs. ER+ class are predominately present (60.0%) in ER- (poorer prognosis class) while a different set of 6 SNVs (40.0%) is identified in ER+ (better prognosis class). In PR- vs. PR+ comparison, 6 SNVs are significantly present (60.0%) in PR- (poorer prognosis class), compared to 4 (40.0%) in PR+ (better prognosis class). Other classes with poorer prognosis, all have higher number of deleterious mutations, with 4 SNVs (66.7%) in HER2+, 7 (87.5%) in Grade III, and 10 (100.0%) in Stage III. Besides, in Grade II vs. Grade III, Fig. 3 also shows an increased risk of having hypertension comorbidity in Grade III patients because of the higher mutation rate for ADD1 gene in this class (16% in Grade II vs. 76.9% in Grade III).

**Table 7:** Differentially occurring SNVs with deleterious mutations in domain regions.

**SNP in ER comparison**	**ER-** ^a^	**ER+** ^b^	**OR** ^c^	**p-value** ^d^	**dbSNP ID**	**AA change**	**Functional Domain**
SLC24A1_chr15_65916527_65916527_A_T	23/27	13/40	11.94	2.11E-05	rs3743171	p.T37S	PfamB PB047652
CSN3_chr4_71114956_71114956_G_T	15/27	7/40	5.89	1.59E-03	rs1048152	p.R110L	Kappa casein
ERBB2_chr17_37884037_37884037_C_G	8/27	26/40	0.23	6.24E-03	rs1058808	p.P1140A	PfamB PB015832
PPP2R4_chr9_131909736_131909736_C_T	11/27	2/40	13.06	4.29E-04	rs2480452	p.S287L	Phosphotyrosyl phosphate activator (PTPA) protein
DPP3_chr11_66276576_66276576_G_A	7/27	28/40	0.15	1.41E-03	rs12421620	p.E690K	Peptidase family M49
KRT74_chr12_52966428_52966428_G_C	12/27	5/40	5.60	4.53E-03	rs11170177	p.N165K	Intermediate filament
GPR157_chr1_9165685_9165685_G_A	5/27	24/40	0.15	1.01E-03	rs72637739	p.R218C	Secretin receptor family
FAM209B_chr20_55111364_55111364_A_C	12/27	4/40	7.20	2.63E-03	rs2296129	p.E129A	FAM209 family
**SNP in PR comparison**	**PR-** ^**a**^	**PR+** ^**b**^	**OR** ^**c**^	**p-value** ^**d**^	**dbSNP ID**	**AA change**	**Functional Domain**
KIAA1377_chr11_101832590_101832590_C_A	15/30	4/37	8.25	7.84E-04	rs11225089	p.S275Y	Susceptibility to monomelic amyotrophy
CPN1_chr10_101829514_101829514_C_T	10/30	1/37	18.00	1.57E-03	rs61751507	p.G178D	Zinc carboxypeptidase (Peptidase_M14)
RBM46_chr4_155719189_155719189_T_G	8/30	0/37	0.36/0	8.97E-04	rs79167802	p.I126M	RNA recognition motif (RRM_1)
DPP3_chr11_66276576_66276576_G_A	9/30	26/37	0.18	1.41E-03	rs12421620	p.E690K	Peptidase family M49
HKR1_chr19_37854040_37854040_G_A	12/30	4/37	5.50	8.63E-03	rs2921563	p.R448H	Zinc-finger double domain (zf-H2C2_2)
**SNP in HER2 comparison**	**HER2-** ^**a**^	**HER2+** ^**b**^	**OR** ^**c**^	**p-value** ^**d**^	**dbSNP ID**	**AA change**	**Functional Domain**
CENPJ_chr13_25486911_25486911_G_T	5/42	4/8	0.14	2.64E-02	rs9511510	p.P85T	PfamB PB003077
GABRE_chrX_151138179_151138179_A_C	40/42	4/8	20.00	3.94E-03	rs1139916	p.S102A	Neurotransmitter-gated ion-channel ligand binding domain
SP4_chr7_21469504_21469504_C_G	3/42	4/8	0.08	8.54E-03	rs139491266	p.L241V	PfamB PB022696
**SNP in Grade comparison**	**GradeII** ^**a**^	**GradeIII** ^**b**^	**OR** ^**c**^	**p-value** ^**d**^	**dbSNP ID**	**AA change**	**Functional Domain**
ANKRD32_chr5_94030818_94030818_G_T	0/25	4/13	0.00	9.69E-03	rs76504036	p.C993F	PfamB PB101142
GFM2_chr5_74037386_74037386_T_A	2/25	5/13	0.14	3.41E-02	rs16872235	p.S300C	Elongation factor Tu GTP binding domain
**SNP in Stage comparison**	**StageII** ^**a**^	**StageIII** ^**b**^	**OR** ^**c**^	**p-value** ^**d**^	**dbSNP ID**	**AA change**	**Functional Domain**
LPPR2_chr19_11473358_11473358_C_G	1/70	5/18	0.04	1.14E-03	rs11540666	p.T253S	PAP2 superfamily
PRCP_chr11_82564294_82564294_T_G	5/70	8/18	0.10	4.80E-04	rs2229437	p.E112D	Serine carboxypeptidase S28
GSTO1_chr10_106022789_106022789_C_A	13/70	11/18	0.15	7.33E-04	rs4925	p.A140D	Glutathione S-transferase, C-terminal domain
PLEKHG6_chr12_6421495_6421495_G_A	26/70	15/18	0.12	5.23E-04	rs740842	p.A35T	PfamB PB015161
AGL_chr1_100358103_100358103_C_T	5/70	5/18	0.20	2.72E-02	rs3753494	p.P1051S	Amylo-alpha-1,6-glucosidase
AGL_chr1_100361925_100361925_G_A	20/70	10/18	0.32	4.93E-02	rs2230307	p.G1115R	Amylo-alpha-1,6-glucosidase
MMP20_chr11_102482504_102482504_T_G	3/70	5/18	0.12	8.03E-03	rs17099008	p.I169L	Matrixin (Peptidase_M10)

^a^ SNV mutate ratio in ER-, PR-, HER2-, Grade II, and Stage II. (number of patients with the mutation in the class/total number of patients in the class)

^b^ SNV mutate ratio in ER+, PR+, HER2+, Grade III, and Stage III. (number of patients with the mutation in the class/total number of patients in the class)

^c^ OR: Odd ratio (ER-/ER+; PR-/PR+; HER2-/HER2+; GradeII/GradeIII; StageII/StageIII)

^d^ Fisher’s exact test

The deleterious mutation shown in Table 7 for ERBB2 is rs1058808. A previous study has shown that rs1058808 may be associated with higher Body Mass Index (BMI) for high risk of endometrial cancer [85]. Although the association between this SNV and the risk of breast cancer is not identified as statistically significant in these studies [86,87], our results show that this mutation is preferably present in the ER+ compared to the ER- subtype (odd ratio 0.23, p = 0.00624). Another mutation, rs2480452 in PPP2R4 is predominantly present in the ER- subtype (40.7% in ER- vs. 5% in ER+ with odd ratio of 13.06, p = 4.29×10^-4^). Our protein stability analysis also suggests that this mutation is destabilizing PPP2R4 protein (Table 8). As mutations of PPP2R4 are significant in the pathogenesis of breast cancer [41], especially in different ER status patients, the downstream effect of this SNV on protein stability is further investigated in the next section.

**Table 8:** Pfam and Panther motif analysis for breast cancer related mutated genes and overall impact of mutation in protein stability.

**Gene Symbol**	**dbSNP**	**Protein**	**AA change**	**Pfam** ^a^	**HMMPanther** ^b^	**Impact of mutation** ^c^
CPN1	rs61751507	P15169	p.G178D	Peptidase M14 (PF00246)	Protease M14 Carboxypeptidase (PTHR11532)	Destabilizing
AGL	rs2230307	P35573	p.G1115R	GDE_C (PF06202)	Glycogen Debranching Enzyme (PTHR10569)	Destabilizing
PPP2R4	rs2480452	Q15257	p.S287L	PTPA (PF03095)	Serine/Threonine-Protein Phosphatase 2A Regulatory Subunit B (PTHR10012)	Destabilizing
GPR157	rs72637739	Q5UAW9	p.R218C	7tm_2 (PF00002)	G Protein-Coupled Receptor 157 (PTHR23112)	Destabilizing
GFM2	rs16872235	Q969S9	p.S300C	GTP_EFTU (PF00009)	Translation elongation factor G (PTHR23115)	Stabilizing
CENPJ	rs9511510	Q9HC77	p.P85T	——	T complex protein 10 (PTHR10331)	Destabilizing
DPP3	rs12421620	Q9NY33	p.E690K	PeptidaseM49 (PF03571)	Dipeptidyl peptidase III (PTHR23422)	Destabilizing
ANKRD32	rs11225089	Q9BQI6	p.C993F	——	——	Stabilizing
KIAA1377	rs61751507	Q9P2H0	p.S275Y	K1377 (PF15352)	Pthr31191 family not named (PTHR31191)	Destabilizing

^a^ Pfam ID (Pfam accession ID)

^b^ Panther family (Panther accession ID)

^c^ Test scores for stabilizing/destabilizing are shown in S5 Table

Table 7 shows SNVs that have significantly different occurrence frequency between different breast classes. For example, rs11225089, rs61751507, and rs79167802 occur more frequently in the PR- than PR+ class; rs1139916 occur more frequently in the HER2- than HER2+ class; rs76504036 occur more frequently in the grade III than in grade II class; rs11540666 and rs2229437 occur more frequently in the stage III than in stage II class. These SNVs might be related to tumor evolution and contribute to different prognosis of breast cancer subtypes.

There are some SNVs that are differentially occurring between the comparison groups but not present in the functional domain regions of proteins (S3 Table). However, it is possible that these SNVs are present in the inter-domain or loop regions, but still have an effect on the structure of protein or otherwise affect a protein’s ability to bind and interact with other proteins.

### Protein stability analysis

For feasibility, we selected 9 relatively rare occurring SNVs from Table 7 to analyze the consequences of point mutations on protein stability. We carried out hmmpfam/hmmpanther motif search with iprscan, to assess if the SNVs are part of the functional motif or domain region (with high confidence at an E-value < = 10^-4^) (Table 8). Then, we compared two protein structure prediction tools, I-TASSER and RaptorX, using the known PDB structure of DPP3 (PDB ID- 3FVY) to determine which method is more reliable and accurate for protein structure prediction. The Root-Mean-Square Deviations (RMSD) of atomic positions between the known DPP3 PDB structure and the I-TASSER or RaptorX predicted models are 0.45 and 7.1, respectively, indicating that I-TASSER is performing far better than RaptorX. We repeated the structure prediction twice for each protein, in order to check if we can get the same structure for each run or not. I-TASSER always gave the same result while RaptorX often gave slightly different results for several proteins. Thus we used I-TASSER for further analysis of all proteins.

**Fig 4:**
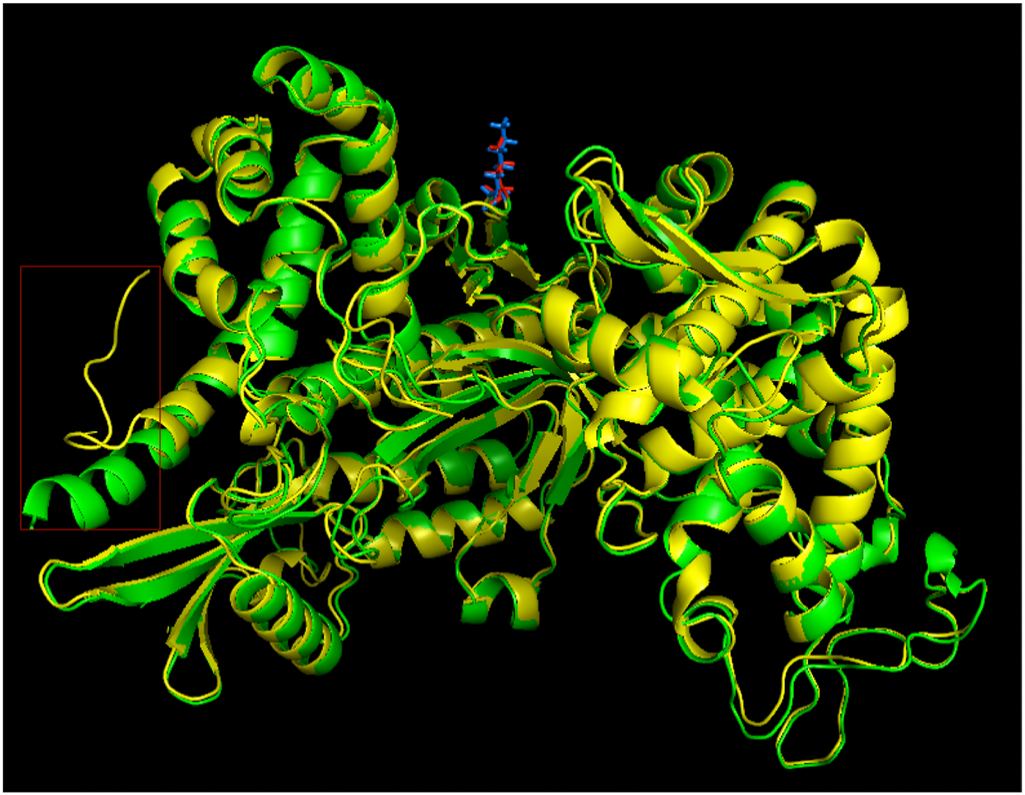
Superimposed structures of normal (green) and mutated (yellow) DPP3 protein chains. Amino acid change at 690th position for DPP3 leads to the structural changes at the C-terminus (in red square) region of the mutant protein. Normal residue (E) at 690th position is shown in blue and the mutated residue (K) is shown in red.

Further, we analyzed the impact of point mutations (SNVs) on protein stability by using I-Mutant 2.0, PopMusic2.1 and CUPSAT tools (S5 Table). Our results suggest that 7 out of 9 SNVs tested have destabilizing effect on proteins. In contrast, the other two SNVs (present in GFM2 and ANKRD32 proteins) have a stabilizing effect (means no significant change to structure or function) after mutation (Table 8) (Fig. 4). In mutated DPP3 protein, negatively charged Glutamate residue (E) got replaced with positively charged Lysine (K) at position 690. Structure analysis of DPP3 suggests that mutant protein has almost similar structure to normal protein, except that the C-terminus has its helix structure changed to a loop structure because of the point mutation (Fig. 4). It has been reported that the C-terminal structure of this protein can play a big role in substrate binding in DPP3 [88]. As the mutation occurs close to the substrate binding residues, K666 and R669 [88], we hypothesize that the altered structure at C-terminus affects substrate binding and consequently alters protein function.

## Conclusions

Breast cancers exhibit highly heterogeneous molecular profiles, which often reflect their distinct prognosis. Although gene expression profiles have been widely used for the classification and targeted treatment of breast cancers, DNA mutational profiles—owing to their stability of detection—are more advantageous in developing biomarkers. In this study, we attempt to detect the genetic mutations (at gene- and nucleotide-level) that are significantly different across different breast cancer classes, by performing a large-scale analysis of 98 breast cancer exome sequencing datasets. We proposed a method for scoring the deleteriousness of mutated genes and identified differentially mutated genes (DMGs) and SNVs from five breast cancer comparison classes (ER+ vs. ER-, PR+ vs. PR-, HER2+ vs. HER2-, grade II vs. grade III, and stage II vs. stage III). We have identified many DMGs such as ERBB2, EPS8, PPP2R4, KIAA0922, SP4, CENPJ, PRCP and SELP, whose mutational loads match with experimentally or clinically verified breast cancer prognosis. We also identified some category 2 genes such as ARL6IP5, RAET1E, and ANO7 that could be crucial for breast cancer development and prognosis (S1 File). Interestingly, the majority of DMGs have higher deleterious mutation scores in the classes with poor prognosis (ER-, PR-, HER2+, grade III, and stage III), which suggests that the deleterious gene mutations are gradually accumulated with the progression of cancer.

Then, we identified some SNVs such as rs1058808, rs2480452, rs61751507, rs79167802, rs11540666, and rs2229437 that potentially influence protein functions and have significantly different occurrence frequency in the populations of different breast cancer comparison groups. Protein structure analysis also suggests that many of the SNVs identified in this study could alter the protein stability and structure, and those SNVs might be associated with cancer evolution and affect prognosis of breast cancers. Some genes and SNVs we identified are worthy of further experimental investigation and verification.

## Supporting Information

S1 File Supplementary literatures for category 2–4 DMGs. Literatures are listed for each class of comparison. Tables in the file were sorted based on categories.
(DOCX)

S1 Table Clinical information of all 103 breast cancer samples. Information includes ID for this study (ID), dbGap subject ID (dbGap SubjID), submitted subject ID (SUBJID), Age, Gender, Primary Disease, Expression Subtype, Country, ER status, PR status, HER 2 status, tumor stage (Stage), tumor grade (Grade), Menopausal Status, Histology, and whether it is used in this study (In the study).
(XLSX)

S2 Table Deleterious mutation score matrix for filtered 3,826 genes in 98 breast cancer samples. Genes that have deleterious mutations present in less than 5 (out of 98) samples have been filtered out to obtain 3,826 genes. Deleterious scores were calculated using the scoring function described in method section.
(XLSX)

S3 Table All the deleterious SNVs identified from five two-class comparison. Differentially mutated genes among ER+ vs. ER-, PR+ vs. PR-, HER2+ vs. HER2-, grade II vs. grade III, and stage II vs. stage III are listed, along with the occurrences and functional domain information.
(XLSX)

S4 Table Comparison of mutation deleteriousness scores between CONDEL and SIFT for all SNVs from DMGs. (XLSX)

S5 Table Protein stability test results for selected SNVs using I-MUTANT 2.0, PopMusic 2.1 and CUPSAT. A mutation is defined as destabilizing/stabilizing if at least two tools give the same prediction result.
(XLSX)
