# Supplementary material for: Exome Analysis Reveals Differentially Mutated Gene Signatures of Stage, Grade and Subtype in Breast Cancers
Source: PLoS One. 2015 Mar 24;10(3):e0119383. doi: 10.1371/journal.pone.0119383 (PMC4372331; doi:10.1371/journal.pone.0119383)
Supplement: S1 File — Literatures are listed for each class of comparison. Tables in the file were sorted based on categories. (DOCX) [file pone.0119383.s001.docx]

**Literatures support for differentially mutated genes between hormone receptor positive and negative breast cancer subtypes in Category 2-4.**

Table 2. Differentially mutated genes between ER+ and ER- breast cancer subtypes

| **Gene Symbol** | **P-value** | **FDR** ^a^ | **Mean of mutation score in ER-** | **Mean of mutation score in ER+** | **FC** ^b^ | **Gene Name** | **Category ^c^** |
| --- | --- | --- | --- | --- | --- | --- | --- |
| CSN3 | 7.06E-05 | 0.090 | 0.61 | 0.16 | 3.75 | casein kappa | 1 |
| ERBB2 | 1.57E-04 | 0.099 | 0.32 | 0.81 | 0.40 | v-erb-b2 erythroblastic leukemia viral oncogene homolog 2, neuro/glioblastoma derived oncogene homolog (avian) | 1 |
| PPP2R4 | 2.09E-04 | 0.099 | 0.44 | 0.04 | 10.40 | protein phosphatase 2A activator, regulatory subunit 4 | 1 |
| CAPZA2 | 4.02E-04 | 0.128 | 0.75 | 0.33 | 2.24 | capping protein (actin filament) muscle Z-line, alpha 2 | 1 |
| SKOR1 | 7.56E-04 | 0.181 | 0.41 | 0.80 | 0.51 | SKI family transcriptional corepressor 1 | 1 |
| ARL6IP5 | 1.72E-04 | 0.099 | 0.40 | 0.04 | 9.39 | ADP-ribosylation-like factor 6 interacting protein 5 | 2 |
| RAET1E | 2.28E-04 | 0.099 | 0.63 | 0.20 | 3.14 | retinoic acid early transcript 1E | 2 |
| DPP3 | 2.54E-04 | 0.099 | 0.26 | 0.70 | 0.38 | dipeptidyl-peptidase 3 | 2 |
| OR1J2 | 4.04E-05 | 0.090 | 0.33 | 0.00 | INF | olfactory receptor, family 1, subfamily J, member 2 | 3 |
| OR52E6 | 1.68E-04 | 0.099 | 0.86 | 0.43 | 2.00 | olfactory receptor, family 52, subfamily E, member 6 | 3 |
| GPR157 | 5.57E-04 | 0.142 | 0.18 | 0.58 | 0.30 | G protein-coupled receptor 157 | 3 |
| SLC24A1 | 6.30E-05 | 0.090 | 0.85 | 0.34 | 2.46 | solute carrier family 24 (sodium/potassium/calcium exchanger), member 1 | 4 |
| KRT74 | 2.59E-04 | 0.099 | 0.59 | 0.18 | 3.37 | keratin 74 | 4 |
| DIS3L | 2.85E-04 | 0.099 | 0.49 | 0.10 | 4.97 | DIS3 mitotic control homolog (S. cerevisiae)-like | 4 |
| OC90 | 4.68E-04 | 0.138 | 0.26 | 0.00 | INF | otoconin 90 | 4 |
| DYNC2LI1 | 5.56E-04 | 0.142 | 0.37 | 0.80 | 0.46 | dynein, cytoplasmic 2, light intermediate chain 1 | 4 |
| GLYATL3 | 8.67E-04 | 0.192 | 0.44 | 0.82 | 0.54 | chromosome 6 open reading frame 140 | 4 |
| FAM209B | 9.02E-04 | 0.192 | 0.43 | 0.10 | 4.44 | family with sequence similarity 209, member B | 4 |

^a^ FDR: False Discovery Rate

^b^ FC: fold change (ER-/ER+); INF: infinite

^c^ Category 1: directly related to breast cancer;

Category 2: related to other types of cancer, but not to breast cancer;

Category 3: other members of the same family (but not by itself) are related to cancer;

Category 4: not belonging to any of the former three categories.

*All the above notations apply to Table 3, 4, 5 and 6.

Table 3. Differentially mutated genes between PR+ and PR- breast cancer subtypes

| **Gene Symbol** | **p-value** | **FDR** ^a^ | **Mean of mutation score in PR-** | **Mean of mutation score in PR+** | **FC** ^b^ | **Gene Name** | **Category** ^c^ |
| --- | --- | --- | --- | --- | --- | --- | --- |
| SKOR1 | 1.14E-04 | 0.297 | 0.40 | 0.84 | 0.48 | SKI family transcriptional corepressor 1 | 1 |
| CPN1 | 5.47E-04 | 0.425 | 0.32 | 0.03 | 11.27 | carboxypeptidase N, polypeptide 1 | 1 |
| ARID5A | 1.00E-03 | 0.425 | 0.37 | 0.06 | 6.49 | AT rich interactive domain 5A (MRF1-like) | 1 |
| DPP3 | 7.74E-04 | 0.425 | 0.30 | 0.70 | 0.42 | dipeptidyl-peptidase 3 | 2 |
| OR1J2 | 2.14E-04 | 0.297 | 0.30 | 0.00 | INF | olfactory receptor, family 1, subfamily J, member 2 | 3 |
| HKR1 | 8.95E-04 | 0.425 | 0.50 | 0.14 | 3.60 | GLI-Kruppel family member HKR1 | 3 |
| KIAA1377 | 2.33E-04 | 0.297 | 0.56 | 0.11 | 5.01 | KIAA1377 | 4 |
| RBM46 | 5.91E-04 | 0.425 | 0.26 | 0.00 | INF | RNA binding motif protein 46 | 4 |
| WDR87 | 8.72E-04 | 0.425 | 0.14 | 0.54 | 0.26 | WD repeat domain 87 | 4 |

*All the above notations apply to Table 3, 4, 5 and 6.

ARL6IP5 is involved in the regulation of many cellular processes including apoptosis, migration and invasion [[1](#_ENREF_1)]. It has been reported to be a prognostic factor for overall survival and tumor progression factor for esophageal squamous cell carcinoma [[1](#_ENREF_1)] and gastric cancer [[2](#_ENREF_2)]. Our results show that the gene has a higher deleterious mutation score in ER- breast cancers than in ER+ breast cancers (p-value=1.72×10^-4^), suggesting that it might be a prognostic factor for breast cancers.

For RAET1E and DPP3, one study reported that high level RAET1E expression is associated with a poor prognosis in ovarian cancer [[3](#_ENREF_3)]. DPP3 is a metallopeptidase involved in a number of physiological processes and its expression increase with the histological aggressiveness of human ovarian primary carcinomas [[4](#_ENREF_4),[5](#_ENREF_5)]. Both genes have higher deleterious mutation scores in ER- breast cancers than in ER+ breast cancers (p-value=2.28×10^-4^ and 2.54×10^-4^ for RAET1E and DPP3, respectively), suggesting that they might be associated with prognosis of breast cancers.

OR1J2 and OR52E6 belong to Olfactory Receptor (OR) family. ORs are expressed not only in the sensory neurons of the olfactory epithelium, but also in other tissues. The potential functions for ectopically expressed ORs are largely unknown. One olfactory receptor, OR51E2, plays a role in cancerogenesis by reducing the growth of prostate cancer cells [[6](#_ENREF_6)]. Besides, abnormally diminished olfactory function was also reported in women with ER+ breast cancer [[7](#_ENREF_7)].

GPR157 is one type of the G-protein coupled receptors (GPCRs). Previous study suggested that GPCRs might be also involved in hormone-refractory cancers [[8](#_ENREF_8)]. One of the GPCRs, GPR30, has been found to be overexpressed in ER+ breast cancer cell lines [[9](#_ENREF_9)]. Recent study shows that GPR30 acts as an inhibitor of tamixifen resistance in hormone-dependent breast cancer. Direct evidence that GPR30 binds estrogens and has the signal transduction characteristics of a membrane ER has been obtained from the studies with transfected HEK293 cells, which lack both nuclear ER-α and ER-β [[10](#_ENREF_10),[11](#_ENREF_11)]. Our results suggest that GPR157 might be another GPCR that is involved in hormone-refractory cancer.

HKR1 belongs to GLI-Kruppel zinc finger family. One of the GLI-Kruppel family zinc finger protein, GLI1, is an established oncogene [[12](#_ENREF_12)] that promotes metastasis in ER-α negative breast cancer cell lines [[13](#_ENREF_13)] and its expression in ER-α negative [[14](#_ENREF_14)] and triple negative breast cancers is indicative of a poor prognosis of breast cancer [[15](#_ENREF_15),[16](#_ENREF_16),[17](#_ENREF_17)]. Evidence shows that GLI1 encodes a transcription factor that is involved in hedgehog/GLI signal pathway, one pathway that plays an important role in the development and progression of breast cancer [[14](#_ENREF_14)].

KRT74 encodes a keratin protein responsible for structural integrity of epithelial cells and specifically expressed in the inner root sheath of hair follicles [[18](#_ENREF_18)]. The gene has a higher deleterious mutation score in ER- breast cancers than in ER+ breast cancers (p-value=2.59×10^-4^).

**Literatures support for differentially mutated genes between HER2+ and HER2- breast cancer subtypes in Category 2-4.**

Table 4. Differentially mutated genes between HER2+ and HER2- breast cancer subtypes

| **Gene Symbol** | **p-value** | **FDR** ^a^ | **Mean of mutation score in HER2-** | **Mean of mutation score in HER2+** | **FC** ^b^ | **Gene Name** | **Category** ^c^ |
| --- | --- | --- | --- | --- | --- | --- | --- |
| BCAR1 | 1.06E-05 | 0.020 | 0.00 | 0.37 | 0.00 | similar to breast cancer anti-estrogen resistance 1; breast cancer anti-estrogen resistance 1 | 1 |
| CENPJ | 2.59E-04 | 0.248 | 0.56 | 1.46 | 0.38 | centromere protein J | 1 |
| EPS8 | 4.61E-04 | 0.294 | 0.03 | 0.37 | 0.08 | epidermal growth factor receptor pathway substrate 8 | 1 |
| KIAA0922 | 6.15E-04 | 0.332 | 0.00 | 0.25 | 0.00 | KIAA0922 | 1 |
| SP4 | 9.61E-04 | 0.380 | 0.07 | 0.48 | 0.15 | Sp4 transcription factor | 1 |
| GABRE | 3.89E-04 | 0.294 | 0.93 | 0.48 | 1.95 | gamma-aminobutyric acid (GABA) A receptor, epsilon | 3 |
| TTC7A | 1.06E-05 | 0.020 | 0.00 | 0.38 | 0.00 | tetratricopeptide repeat domain 7A | 3 |
| DIS3L | 1.00E-03 | 0.380 | 0.07 | 0.50 | 0.14 | DIS3 mitotic control homolog (S. cerevisiae)-like | 4 |
| ZCWPW1 | 1.39E-04 | 0.177 | 0.04 | 0.49 | 0.09 | zinc finger, CW type with PWWP domain 1 | 4 |
| ZNF233 | 6.95E-04 | 0.332 | 0.12 | 0.62 | 0.20 | zinc finger protein 233 | 4 |

*All the above notations apply to Table 3, 4, 5 and 6.

TTC7A belongs to tetratricopeptide repeat (TPR) domain. TTC7A is a member of the TPR repeat protein family known to interact with other proteins, to facilitate transport, and to act as chaperone or scaffolding proteins [[19](#_ENREF_19)]. One of the TPR, TTC9A, was identified as a steroid hormone-regulated gene in various breast cancer cells [[20](#_ENREF_20)]. TTC9A has been suggested to play a role in cancer cell invasion and metastasis [[21](#_ENREF_21)]. GABRE encodes the gamma-aminobutyric acide (GABA) A receptor. One gene from the same family, gamma-aminobutyric acide A receptor pi (GABRP), has been reported to be progressively down-regulated with tumor-progression, and could be used as a prognostic marker for breast cancer [[22](#_ENREF_22)].

**Literatures support for differentially mutated genes between Grade II and Grade III breast cancer subtypes in Category 2-4.**

Table 5. Differentially mutated genes between Grade II and Grade III breast cancer classes

| **Gene Symbol** | **p-value** | **FDR** ^a^ | **Mean of mutation score in Grade II** | **Mean of mutation score in Grade III** | **FC** ^b^ | **Gene Name** | **Category** ^c^ |
| --- | --- | --- | --- | --- | --- | --- | --- |
| SELP | 6.73E-05 | 0.230 | 0.00 | 0.45 | 0.00 | selectin P (granule membrane protein 140kDa, antigen CD62) | 1 |
| ANO7 | 6.32E-04 | 0.460 | 0.16 | 0.68 | 0.24 | anoctamin 7 | 2 |
| ANKRD18B | 1.22E-04 | 0.230 | 0.12 | 0.69 | 0.18 | ankyrin repeat domain 18B | 3 |
| ANKRD32 | 4.70E-04 | 0.450 | 0.00 | 0.38 | 0.00 | ankyrin repeat domain 32 | 3 |
| THAP8 | 8.19E-04 | 0.460 | 0.51 | 0.00 | INF | THAP domain containing 8 | 3 |
| ADD1 | 3.63E-04 | 0.450 | 0.31 | 1.33 | 0.23 | adducin 1 (alpha) | 4 |
| GFM2 | 8.37E-04 | 0.460 | 0.12 | 0.61 | 0.20 | G elongation factor, mitochondrial 2 | 4 |

*All the notations are the same as in Table 2. No clinic information for the tumor grade was given for breast cancer primary tumor sample. Therefore, differential gene expression statistics are not available for Grade II vs. Grade III

ANO7, also known as New Gene Expressed in Prostate (NGEP), is a prostate-specific polytopic membrane protein. Studies have shown that NGEP protein is widely expressed in low-grade to high-grade prostate adenocarcinomas as well as benign prostate tissues, and the intensity of expression is inversely proportional to the level of malignancy [[23](#_ENREF_23)]. Our results show that the gene has a higher deleterious mutation score in Grade III breast cancers than in Grade II breast cancers (p-value=6.32×10^-4^), suggesting that the degree of deleterious mutations is proportional to the level of malignancy. Both ANKRD18B and ANKRD32 encode the proteins belonging to the ankyrin repeats-containing cofactor family.  Ankyrin repeat is one of the most widely existing protein motifs in nature that exclusively mediates protein-protein interactions, some of which are directly involved in the development of human cancer and other diseases [[24](#_ENREF_24)].  ANKRD32 encodes a protein containing the carboxyl-terminal domain (BRCT) which is the signature domain of breast cancer gene 1 (BRCA1). ANKRD32 is located in chromosome 5q15, which has been shown to be a hotspot for rearrangements in p53-negative tumors [[25](#_ENREF_25)]. Our results show that the gene has a higher deleterious mutation score in Grade III breast cancers than in Grade II breast cancers (p-value=4.70×10^-4^), suggesting that its mutations evolve with the progression of breast cancers. THAP8 encodes a protein that contains the THAP domain, a novel protein motif found with similarity to the DNA-binding domain of P element transposase. Although the role of THAP8 in breast cancer is unclear, another THAP domain containing gene, THAP10, is one part of a bidirectional gene pair on the long arm of chromosome 15 that is regulated by estrogen and may play a role in breast cancer [[26](#_ENREF_26)].

**Literatures support for differentially mutated genes between Stage II and Stage III breast cancer subtypes in Category 2-4.**

Table 6. Differentially mutated genes between Stage II and Stage III breast cancer classes

| **Gene Symbol** | **p-value** | **FDR** ^a^ | **Mean of mutation score in Stage II** | **Mean of mutation score in Stage III** | **FC** ^b^ | **Gene Name** | **Category** ^c^ |
| --- | --- | --- | --- | --- | --- | --- | --- |
| CPZ | 4.24E-05 | 0.055 | 0.01 | 0.39 | 0.04 | carboxypeptidase Z | 1 |
| LPPR2 | 4.29E-05 | 0.055 | 0.01 | 0.26 | 0.05 | lipid phosphate phosphatase-related protein type 2 | 1 |
| PRCP | 1.44E-04 | 0.138 | 0.08 | 0.43 | 0.19 | prolylcarboxypeptidase (angiotensinase C) | 1 |
| UNC45A | 5.03E-04 | 0.297 | 0.01 | 0.23 | 0.06 | unc-45 homolog A (C. elegans) | 1 |
| PLEKHG6 | 5.44E-04 | 0.297 | 0.38 | 0.82 | 0.46 | pleckstrin homology domain containing, family G (with RhoGef domain) member 6 | 1 |
| MMP20 | 7.99E-04 | 0.339 | 0.06 | 0.33 | 0.17 | matrix metallopeptidase 20 | 2 |
| CDH26 | 4.21E-05 | 0.055 | 0.01 | 0.28 | 0.05 | cadherin-like 26 | 3 |
| GSTO1 | 2.93E-04 | 0.224 | 0.21 | 0.66 | 0.32 | glutathione S-transferase omega 1 | 3 |
| AGL | 6.42E-04 | 0.307 | 0.37 | 0.83 | 0.44 | amylo-1, 6-glucosidase, 4-alpha-glucanotransferase | 4 |
| OGFOD3 | 9.77E-04 | 0.374 | 0.07 | 0.39 | 0.18 | 2-oxoglutarate and iron-dependent oxygenase domain containing 3 | 4 |

*All the above notations apply to Table 3, 4, 5 and 6.

MMP20 plays a role in lymph node metastasis in laryngeal squamous cell carcinoma [[27](#_ENREF_27)]. Although there is no direct evidence that supports the role of this gene in breast cancer, our results indicate that it might play a role in breast cancer progress. CDH26 belongs to Cadherin superfamily and plays an important role in cell adhesion [[28](#_ENREF_28)]. Although the function of CDH26 is not clear, one of the gene from Cadherin superfamily, CDH1, also known as E-cadherin, has been reported to be involved in tumor progression and metastasis in many cancers [[29](#_ENREF_29),[30](#_ENREF_30),[31](#_ENREF_31),[32](#_ENREF_32),[33](#_ENREF_33)]. Our results indicate that CDH26 might also be an important gene involved in breast cancer progression. GSTO1 belongs to an omega class glutathione S-transferase (GST) that is involved in the metabolism of xenobiotics [[34](#_ENREF_34)] and carcinogens [[35](#_ENREF_35)]. Polymorphisms in GSTO1 have been shown to be associated with progression of head and neck cancer [[36](#_ENREF_36)]. Some of the polymorphisms are preferably shown in advanced stage of breast cancer [[37](#_ENREF_37)]. Although there is no literature in support of the involvement of AGL with tumor progression and metastasis, our results indicate that the gene might be associated with breast cancer progression.

**References**

1. Shi GZ, Yuan Y, Jiang GJ, Ge ZJ, Zhou J, Gong DJ, et al. (2012) PRAF3 induces apoptosis and inhibits migration and invasion in human esophageal squamous cell carcinoma. BMC Cancer 12: 97.

2. Wang S, Wu X, Chen Y, Zhang J, Ding J, Zhou Y, et al. (2012) Prognostic and predictive role of JWA and XRCC1 expressions in gastric cancer. Clin Cancer Res 18: 2987-2996.

3. McGilvray RW, Eagle RA, Rolland P, Jafferji I, Trowsdale J, Durrant LG (2010) ULBP2 and RAET1E NKG2D ligands are independent predictors of poor prognosis in ovarian cancer patients. Int J Cancer 127: 1412-1420.

4. Simaga S, Babic D, Osmak M, Sprem M, Abramic M (2003) Tumor cytosol dipeptidyl peptidase III activity is increased with histological aggressiveness of ovarian primary carcinomas. Gynecol Oncol 91: 194-200.

5. Shukla AA, Jain M, Chauhan SS (2010) Ets-1/Elk-1 is a critical mediator of dipeptidyl-peptidase III transcription in human glioblastoma cells. FEBS J 277: 1861-1875.

6. Neuhaus EM, Zhang W, Gelis L, Deng Y, Noldus J, Hatt H (2009) Activation of an olfactory receptor inhibits proliferation of prostate cancer cells. J Biol Chem 284: 16218-16225.

7. Lehrer S, Levine E, Bloomer WD (1985) Abnormally diminished sense of smell in women with oestrogen receptor positive breast cancer. Lancet 2: 333.

8. Dorsam RT, Gutkind JS (2007) G-protein-coupled receptors and cancer. Nat Rev Cancer 7: 79-94.

9. Carmeci C, Thompson DA, Ring HZ, Francke U, Weigel RJ (1997) Identification of a gene (GPR30) with homology to the G-protein-coupled receptor superfamily associated with estrogen receptor expression in breast cancer. Genomics 45: 607-617.

10. Thomas P, Pang Y, Filardo EJ, Dong J (2005) Identity of an estrogen membrane receptor coupled to a G protein in human breast cancer cells. Endocrinology 146: 624-632.

11. Kahlert S, Nuedling S, van Eickels M, Vetter H, Meyer R, Grohe C (2000) Estrogen receptor alpha rapidly activates the IGF-1 receptor pathway. J Biol Chem 275: 18447-18453.

12. Nagai S, Nakamura M, Yanai K, Wada J, Akiyoshi T, Nakashima H, et al. (2008) Gli1 contributes to the invasiveness of pancreatic cancer through matrix metalloproteinase-9 activation. Cancer Sci 99: 1377-1384.

13. Kwon YJ, Hurst DR, Steg AD, Yuan K, Vaidya KS, Welch DR, et al. (2011) Gli1 enhances migration and invasion via up-regulation of MMP-11 and promotes metastasis in ERalpha negative breast cancer cell lines. Clin Exp Metastasis 28: 437-449.

14. Fiaschi M, Rozell B, Bergstrom A, Toftgard R (2009) Development of mammary tumors by conditional expression of GLI1. Cancer Res 69: 4810-4817.

15. Kubo M, Nakamura M, Tasaki A, Yamanaka N, Nakashima H, Nomura M, et al. (2004) Hedgehog signaling pathway is a new therapeutic target for patients with breast cancer. Cancer Res 64: 6071-6074.

16. ten Haaf A, Bektas N, von Serenyi S, Losen I, Arweiler EC, Hartmann A, et al. (2009) Expression of the glioma-associated oncogene homolog (GLI) 1 in human breast cancer is associated with unfavourable overall survival. BMC Cancer 9: 298.

17. Xu L, Kwon YJ, Frolova N, Steg AD, Yuan K, Johnson MR, et al. (2010) Gli1 promotes cell survival and is predictive of a poor outcome in ERalpha-negative breast cancer. Breast Cancer Res Treat 123: 59-71.

18. Haines RL, Lane EB (2012) Keratins and disease at a glance. J Cell Sci 125: 3923-3928.

19. White RA, McNulty SG, Nsumu NN, Boydston LA, Brewer BP, Shimizu K (2005) Positional cloning of the Ttc7 gene required for normal iron homeostasis and mutated in hea and fsn anemia mice. Genomics 85: 330-337.

20. Cao S, Iyer JK, Lin V (2006) Identification of tetratricopeptide repeat domain 9, a hormonally regulated protein. Biochem Biophys Res Commun 345: 310-317.

21. Cao S, Ho GH, Lin VC (2008) Tetratricopeptide repeat domain 9A is an interacting protein for tropomyosin Tm5NM-1. BMC Cancer 8: 231.

22. Zafrakas M, Chorovicer M, Klaman I, Kristiansen G, Wild PJ, Heindrichs U, et al. (2006) Systematic characterisation of GABRP expression in sporadic breast cancer and normal breast tissue. Int J Cancer 118: 1453-1459.

23. Mohsenzadegan M, Madjd Z, Asgari M, Abolhasani M, Shekarabi M, Taeb J, et al. (2013) Reduced expression of NGEP is associated with high-grade prostate cancers: a tissue microarray analysis. Cancer Immunol Immunother 62: 1609-1618.

24. Li J, Mahajan A, Tsai MD (2006) Ankyrin repeat: a unique motif mediating protein-protein interactions. Biochemistry 45: 15168-15178.

25. Jain AN, Chin K, Borresen-Dale AL, Erikstein BK, Eynstein Lonning P, Kaaresen R, et al. (2001) Quantitative analysis of chromosomal CGH in human breast tumors associates copy number abnormalities with p53 status and patient survival. Proc Natl Acad Sci U S A 98: 7952-7957.

26. De Souza Santos E, De Bessa SA, Netto MM, Nagai MA (2008) Silencing of LRRC49 and THAP10 genes by bidirectional promoter hypermethylation is a frequent event in breast cancer. Int J Oncol 33: 25-31.

27. Liu Y, Li Y, Liu Z, Zhang L, Anniko M, Duan M (2011) Prognostic significance of matrix metalloproteinase-20 overexpression in laryngeal squamous cell carcinoma. Acta Otolaryngol 131: 769-773.

28. Angst BD, Marcozzi C, Magee AI (2001) The cadherin superfamily: diversity in form and function. J Cell Sci 114: 629-641.

29. Rodriguez FJ, Lewis-Tuffin LJ, Anastasiadis PZ (2012) E-cadherin's dark side: possible role in tumor progression. Biochim Biophys Acta 1826: 23-31.

30. Fan L, Wang H, Xia X, Rao Y, Ma X, Ma D, et al. (2012) Loss of E-cadherin promotes prostate cancer metastasis via upregulation of metastasis-associated gene 1 expression. Oncol Lett 4: 1225-1233.

31. Chen X, Wang Y, Xia H, Wang Q, Jiang X, Lin Z, et al. (2012) Loss of E-cadherin promotes the growth, invasion and drug resistance of colorectal cancer cells and is associated with liver metastasis. Mol Biol Rep 39: 6707-6714.

32. Onder TT, Gupta PB, Mani SA, Yang J, Lander ES, Weinberg RA (2008) Loss of E-cadherin promotes metastasis via multiple downstream transcriptional pathways. Cancer Res 68: 3645-3654.

33. Sawada K, Mitra AK, Radjabi AR, Bhaskar V, Kistner EO, Tretiakova M, et al. (2008) Loss of E-cadherin promotes ovarian cancer metastasis via alpha 5-integrin, which is a therapeutic target. Cancer Res 68: 2329-2339.

34. Seidegard J, Ekstrom G (1997) The role of human glutathione transferases and epoxide hydrolases in the metabolism of xenobiotics. Environ Health Perspect 105 Suppl 4: 791-799.

35. Coles B, Ketterer B (1990) The role of glutathione and glutathione transferases in chemical carcinogenesis. Crit Rev Biochem Mol Biol 25: 47-70.

36. Sanguansin S, Petmitr S, P OC, Pongstaporn W (2012) Association of glutathione S-transferase omega gene polymorphisms with progression of head and neck cancer. Mol Biol Rep 39: 10915-10920.

37. Chariyalertsak S, Purisa W, Sangrajrang S (2009) Role of glutathione S-transferase omega gene polymorphisms in breast-cancer risk. Tumori 95: 739-743.
